# Micromanaging senescence

**DOI:** 10.18632/aging.100158

**Published:** 2010-06-21

**Authors:** Danielle K Jensen, Ashish Lal

**Affiliations:** Immune Disease Institute and Program in Cellular and Molecular Medicine, Children's Hospital Boston, Harvard Medical School, Boston, MA 02115, USA

**Keywords:** 06/16/10; accepted: 06/19/10; published on line: 06/21/10

Normal somatic cells have a limited capacity for division in culture and eventually enter a state of terminal growth arrest termed *replicative senescence* [1]. Cells that fail to undergo senescence divide indefinitely and develop chromosomal aberrations resulting in neoplastic transformation. Senescence is believed to be a tumor suppressor mechanism that ensures that cells permanently exit the cell cycle, rendering them incapable of forming a tumor. Senescent cells do not divide in response to mitogen stimulation, but remain metabolically active and show characteristic changes in cellular morphology. Senescent cells also display specific biochemical properties, such as the expression of a β-galactosidase activity at pH 6 (SA-β-gal) that has long been used as a senescence marker. Senescent cells accumulate as the organism ages and thus the number of SA-β-gal-positive cells increases in older individuals and in individuals with premature aging syndromes. It is well established that senescence is associated with perturbations in gene expression profiles, including increased levels of cell cycle inhibitors such as p16INK4A and p53, and decreased  levels of E2F target genes, and the RNA-binding protein HuR.

MicroRNAs (miRNAs) are a major class of regulatory molecules that inhibit gene expression post-transcriptionally by binding to target mRNAs to promote their degradation and/or inhibit their translation. They have been shown to regulate several processes including cellular proliferation, differentiation and apoptosis. miRNAs are deregulated in cancer and can function as tumor suppressors or oncogenes. Given the link between cancer and senescence, it is not surprising that miRNAs that regulate senescence have Commentary begun to be identified. In fact, global loss of miRNAs has been shown to induce senescence in primary cells [2], suggesting that the miRNA pathway prevents the cells from undergoing senescence. In a physiological context in which normal cells divide and undergo senescence, it is plausible that senescence is achieved by the combined down-regulation of some miRNAs that inhibit senescence and up-regulation of select miRNAs that promote the senescence program. Identifying these senescence-associated miRNAs is therefore essential to understand their roles in senescence and cancer.

In this issue of AGING, Marasa et al., used a highly sensitive genome-wide approach to identify miRNAs that are differentially expressed during replicative senescence of normal human diploid fibroblasts (WI-38 cells). They found that the expression of several miRNAs was altered during senescence, including miR-519, which the authors had previously shown to inhibit the translation of the RNA-binding protein HuR by base-pairing to the coding region of HuR mRNA [3]. Overexpression of miR-519 in early-passage WI-38 cells altered the expression of some senescence-associated proteins: SIRT1 and HuR were down-regulated, whereas p53 and p16INK4A were up-regulated. Down-regulation of HuR in these cells, likely a consequence of base-pairing between miR-519 and HuR mRNA, in turn led to decreased levels of the HuR target SIRT1 mRNA and decreased SIRT1 protein [4]. On the other hand, increased expression of p53 and p16INK4A would likely be an indirect effect of miR-519 overexpression. These changes in gene expression should be sufficient to induce cellular senescence. Indeed, the authors showed that sustained over-expression of miR-519 in early-passage WI-38 cells resulted in a significant decrease in cell number and the induction of senescence. This effect also extended to the highly proliferative and transformed HeLa cells, in which overexpression of miR-519 similarly triggered a senescent phenotype. Given the negative correlation between the abundance of miR-519 and HuR in normal versus tumor tissue and the previous finding that miR-519 reduced tumorigenesis [5], the authors hypothesize that miR-519 represses tumor growth by promoting senescence. They further propose that this effect of miR-519 is partly mediated by down-modulating HuR levels.

The Marasa et al. report identifies an important mechanism by which miR-519 could inhibit tumorigenesis. By down-regulating HuR protein levels, miR-519 would decrease the expression of HuR target genes (such as SIRT1), many of which promote proliferation, invasion and angiogenesis. The findings by Marasa et al., may also have important implications for cancer therapy. Traditional therapies aim to halt cancer by inducing cell differentiation, promoting cell death, or reducing proliferation. By identifying miRNAs associated with replicative senescence, we can envision treatments to induce senescence in tumor cells. miRNA-based therapies are particularly promising because a single miRNA can modulate the expression of numerous genes working in coordination to shut down a biological process. The challenge of specifically delivering the miRNA to tumor cells remains; however, since miRNAs are chemically similar to siRNAs, delivery methods could be employed similar to those currently used for siRNA in mouse models of cancer.

Several important questions remain open for further study. First, is up-regulation of miR-519 essential for cells to undergo senescence? If it is, then stable knockdown of miR-519 in early-passage WI-38 cells should prevent senescence. Second, is miR-519-mediated regulation of HuR important during senescence? Rescue experiments employing an engineered miR-519-insensitive HuR will answer this question. Third, since miR-519 is poorly conserved, what is the evolutionary significance of miR-519-mediated regulation of HuR? Fourth, what mechanisms control the increased expression of miR-519 during senescence? A detailed analysis of the miR-519 gene promoter will help to identify putative transcription factors capable of regulating miR-519 expression during senescence. On the other hand, if miR-519 is regulated post-transcriptionally, identifying RNA-binding proteins that bind to the miR-519 precursor transcripts would provide insight into the regulation of miR-519 abundance.

miRNAs typically down-regulate the expression of hundreds of genes. Although HuR appears to be an important target of miR-519, miR-519 may also lower the expression of many other genes. Recent studies have shown that mRNA degradation is the major mechanism by which mammalian miRNAs inhibit target gene expression [6]. Therefore, over-expressing miR-519 in early-passage WI-38 cells followed by microarray or proteomic analysis will identify many transcripts directly regulated by miR-519. Alternatively, the recently developed Argonaute HITS-CLIP approach [7] could identify cellular transcripts that directly bind to miR-519 *in vivo*. Combining the gene lists generated from these experimental strategies with Gene Ontology and gene network analysis [8] will further elucidate the role of miR-519 in senescence and tumorigenesis.
